# Case report: Clinicopathological and molecular characteristics of pediatric-type follicular lymphoma

**DOI:** 10.3389/fped.2023.1205384

**Published:** 2023-07-19

**Authors:** Beibei Ren, Yu Chen, Xuanye Bai, Jiawen Zheng, Jing Chang, Xiangnan Jiang, Qingxin Xia, He Zhang

**Affiliations:** ^1^ Department of Pathology, Affiliated Cancer Hospital of Zhengzhou University, Zhengzhou, China; ^2^ Henan Medical Key Laboratory of Tumor Pathology and Artificial Intelligence Diagnosis, Zhengzhou, China; ^3^ Pathological Diagnostic Antibody Engineering Research Center of Henan Province, Zhengzhou, China; ^4^ Department of Molecular Pathology, Affiliated Cancer Hospital of Zhengzhou University, Zhengzhou, China; ^5^ Medical Service Office, Affiliated Cancer Hospital of Zhengzhou University, Zhengzhou, China; ^6^ Department of Pathology, Fudan University Shanghai Cancer Center, Shanghai, China

**Keywords:** follicular lymphoma, pediatric type follicular lymphoma, Immunohistochemistry, NGS, molecular

## Abstract

Pediatric-type follicular lymphoma (PTFL) is a rare pediatric-type indolent B-cell lymphoma that clinicopathologically differs from adult lymphoma. Accurate diagnosis of PTFL, which is often challenging, is essential to avoid missed diagnosis, misdiagnosis, and overtreatment. To improve our understanding of PTFL, clinicopathological features, differential diagnosis, and molecular mutation characteristics of four patients of PTFL were analyzed using hematoxylin and eosin staining, immunohistochemistry, polymerase chain reaction, fluorescence *in situ* hybridization (FISH), and next-generation sequencing (NGS). A relevant literature review was also performed. All four PTFL patients were male, with ages of 6, 18, 13, and 15 years, and had St. Jude stage I or III. Microscopic results showed that the structure of the lymph nodes was destroyed; the tumor follicles were enlarged and irregular; medium–large blastoid cells with a consistent shape were visible in tumor follicles, and the nucleus was round or oval; and the “starry sky” pattern was easily observed. Tumor cells expressed CD20, PAX-5, BCL6, and CD10. None of the tumor cells expressed BCL2, CD3, CD5, MUM1, and CyclinD1. CD21 showed dilated growth of a follicular dendritic cell network in tumor follicles. EBER genes were negative in all cases. FISH testing also showed negative *BCL2* gene breaks and *IRF4* gene breaks in all cases. NGS detected 12 related mutant genes, including *KMT2D*, *CD79B*, *GNA13*, *MYD88*, *PCLO*, *TCF3*, *IRF8*, *MAP2K1*, *FOXO1*, *POLE*, *INPP5D*, and *FAT4*. Two of the four patients had an *IRF8* gene mutation, and one patient had a dual mutation of the *MAP2K1* gene. Our study revealed the unique clinicopathological features and molecular mutational characteristics of PTFL, consolidated our understanding of PTFL, and identified other rare mutant genes, which may further contribute to the study of the molecular mechanism and differential diagnosis of PTFL.

## 1. Introduction

Follicular lymphoma (FL) is a mature lymphoid malignancy derived from germinal center B cells, accounting for approximately 22% of all non-Hodgkin's lymphomas. It is common in middle-aged and older people but rare in children and young people. In 2008, the World Health Organization (WHO) classification of lymphoid tissue and hematopoietic system first proposed “pediatric follicular lymphoma” (1). Because of its unique clinical pathology and molecular genetic characteristics, pediatric-type follicular lymphoma (PTFL) was recognized as a distinct entity in the revised 4th edition of the WHO classification of lymphoid neoplasms and was discussed separately in the 5th edition of the WHO classification of lymphoma (2, 3).

The development and maturity of next-generation sequencing (NGS) technology have provided a good platform for the in-depth study of the molecular mechanism associated with the occurrence, development, and prognosis of FL. However, research on NGS-based gene mutations in PTFL is currently limited. In this report, data from four PTFL cases were obtained from the Clinical Pathology Center of the Affiliated Cancer Hospital of Zhengzhou University and the Department of Pathology of Shanghai Cancer Center of Fudan University, and clinicopathological and molecular mutation characteristics were analyzed to improve our understanding of PTFL.

## 2. Materials and methods

### 2.1. Case description

Data from four patients of PTFL were obtained from the Clinical Pathology Center of the Affiliated Cancer Hospital of Zhengzhou University and the Department of Pathology of Shanghai Cancer Center of Fudan University between June 2019 and August 2021. Two pathologists blinded to the molecular results reviewed the cases, and a consensus diagnosis was achieved based on the revised WHO classification of lymphoma (2021 version). One of the specimens was obtained by needle biopsy, and the other two were obtained through excisional biopsy. Relevant clinical and survival data were obtained using the electronic medical record system and through a telephone follow-up. This study was approved by the Institutional Review Boards of the two hospitals and was conducted according to the Declaration of Helsinki.

### 2.2. Hematoxylin and eosin (H&E) and immunohistochemistry (IHC) staining

Tissues were fixed in a 3.7% neutral formaldehyde solution, dehydrated, embedded in paraffin, and sectioned at 4 μm thickness. H&E staining was used to evaluate lymph node biopsies. IHC staining was performed for CD20, CD3, CD5, CD10, BCL2, BCL6, MUM1, CD21, CyclinD1, and Ki-67 as part of the diagnostic workup. Immunostaining was performed on Ventana BenchMark ULTRA following the protocol of the manufacturer. CD20, CD21, CD3, CD5, and CD10 were located in the cell membrane; BCL2 was located in the cell membrane/cytoplasm; and MUM1, BCL6, CyclinD1, and Ki-67 were located in the nucleus.

### 2.3. Epstein–Barr virus–encoded small RNA (EBER) and fluorescence *in situ* hybridization (FISH)

The expression of EBER in PTFL paraffin sections was also detected using Ventana BenchMark ULTRA. Epstein–Barr virus (EBV) *in situ* hybridization was performed using the EBER probe, which was purchased from Roche Diagnostics (Shanghai) Co., Ltd. (Shanghai, China). The experimental procedures were performed according to the instructions of the manufacturer. The nucleus was stained dark blue (positive signal), while the cytoplasm was stained light red. EBV-positive nasopharyngeal carcinoma was selected as positive control. In addition, the *BCL2* and *IRF4* gene rearrangements were detected using fluorescence in situ hybridization (FISH) assays using commercial reagent kits (*BCL2* and *IRF4* Dual Color Break Apart Rearrangement probe, Anbiping Co., Ltd.). A double-color breakage probe contains two probes labeled with green and red fluorescence, respectively. In cells without gene breakage, a yellow fusion signal appears (red and green signals overlap to form a yellow signal), or red and green signals adhere to each other (the interval is less than two signal diameters). In broken genes, a fused yellow signal, a separate green signal, and a separate red signal appear in the nucleus.

### 2.4. B-cell clonality test

Genomic DNA was extracted from formalin-fixed, paraffin-embedded (FFPE) tissue sections using the Maxwell 16 FFPE Tissue LEV DNA Purification Kit and the Maxwell 16 Instrument, following the instructions of the manufacturer. Polymerase chain reaction amplification was performed to detect clonal immunoglobulin (Ig) heavy and/or Ig kappa light chain gene rearrangements, following the BIOMED-2 protocol. The criteria for defining a positive band are as follows: Products generated from diagnostic samples that fall within the valid size range and are at least three times the amplitude of the third largest peak in the polyclonal background are consistent with a positive peak. One or two prominent positive bands within the valid size range are reported as “positive for the detection of clonal immunoglobulin heavy chain or kappa light chain gene rearrangement(s) consistent with the presence of a clonal cell population. In the context of overall diagnostic criteria, clonal cell populations can indicate the presence of hematologic malignancy.” In contrast, an absence of positive bands within the valid size range is reported as “negative for the detection of clonal immunoglobulin heavy chain or kappa light chain gene rearrangement(s).”

### 2.5. Targeted NGS analysis

Targeted NGS was performed on the FFPE tissue samples of the patients using a customized panel of 93 lymphoma-related genes, related to auxiliary diagnosis, prognostic assessment, and targeted drug use of lymphoma. Genomic DNA was extracted from FFPE tissues using the Gene+OncoLym FFPE Kit. Single-nucleotide variants (SNVs) and small insertions and deletions (indels) were detected using an in-house developed pipeline, SNVer and LoFreq. Translocations were detected using Delly and Mantana. Mutations were annotated using SnpEff.

## 3. Results

### 3.1. Clinical characteristics

Case 1 involves a 6-year-old male patient who presented to our hospital for the evaluation of a non-tender mass over the right neck area. A positron emission tomography–computed tomography (CT) scan showed multiple soft tissue nodule shadows in the right cervical regions Ⅱ and Ⅲ with increased metabolism, indicating the presence of malignant lesions of multiple lymph nodes. A biopsy of the right neck was performed for pathological diagnosis. No operation or chemotherapy was performed after the diagnosis using a needle biopsy. The patient underwent follow-up for 28 months without disease progression (Table 1).

**Table 1:** Clinical characteristics of the four patients with PTFL.

Case	Sex	Age (years)	Location	Treatment	Relapse	Progression-free survival (months)
1	Male	6	Right cervical lymph node	Watchful wait	No	28
2	Male	18	Left parotid gland mass	Watchful wait	No	16
3	Male	13	A mass behind the left ear	Watchful wait	No	12
4	Male	15	Right submaxillary lymph node	Watchful wait	No	16

Case 2 involves an 18-year-old male patient who presented to our hospital for the evaluation of a slowly growing and non-tender mass over the left parotid gland area. No systemic symptoms, such as fever, night sweats, and weight loss, were present. A preoperative ultrasound showed a solid mass in the left parotid gland but no abnormal lymphadenopathy in the bilateral neck. There was a solid space-occupying lesion but no abnormal enlargement of lymph nodes. Mass removal with superficial parotidectomy was performed with routine precautions to preserve the facial nerve and the parotid duct. No further treatment following the simple surgical resection was performed. The patient underwent follow-up for 16 months and survived without a tumor (Table 1).

Case 3 involves a 13-year-old male patient who presented with a mass behind the left ear area that had been present for 3 months. No systemic symptoms, such as fever, night sweats, and weight loss, were present. After surgical resection, the patient underwent follow-up for 12 months and survived without a tumor (Table 1).

Case 4 involves a 15-year-old male patient who presented with a non-tender mass on the right neck area that had been present for 2 months. A preoperative CT scan showed that the lymph nodes in the right submaxillary region were swollen, and there were numerous small lymph nodes in the two necks, some of which were slightly larger. After surgical resection, the patient underwent follow-up for 16 months and survived without a tumor (Table 1).

### 3.2. Morphological findings

**Figure 1:**
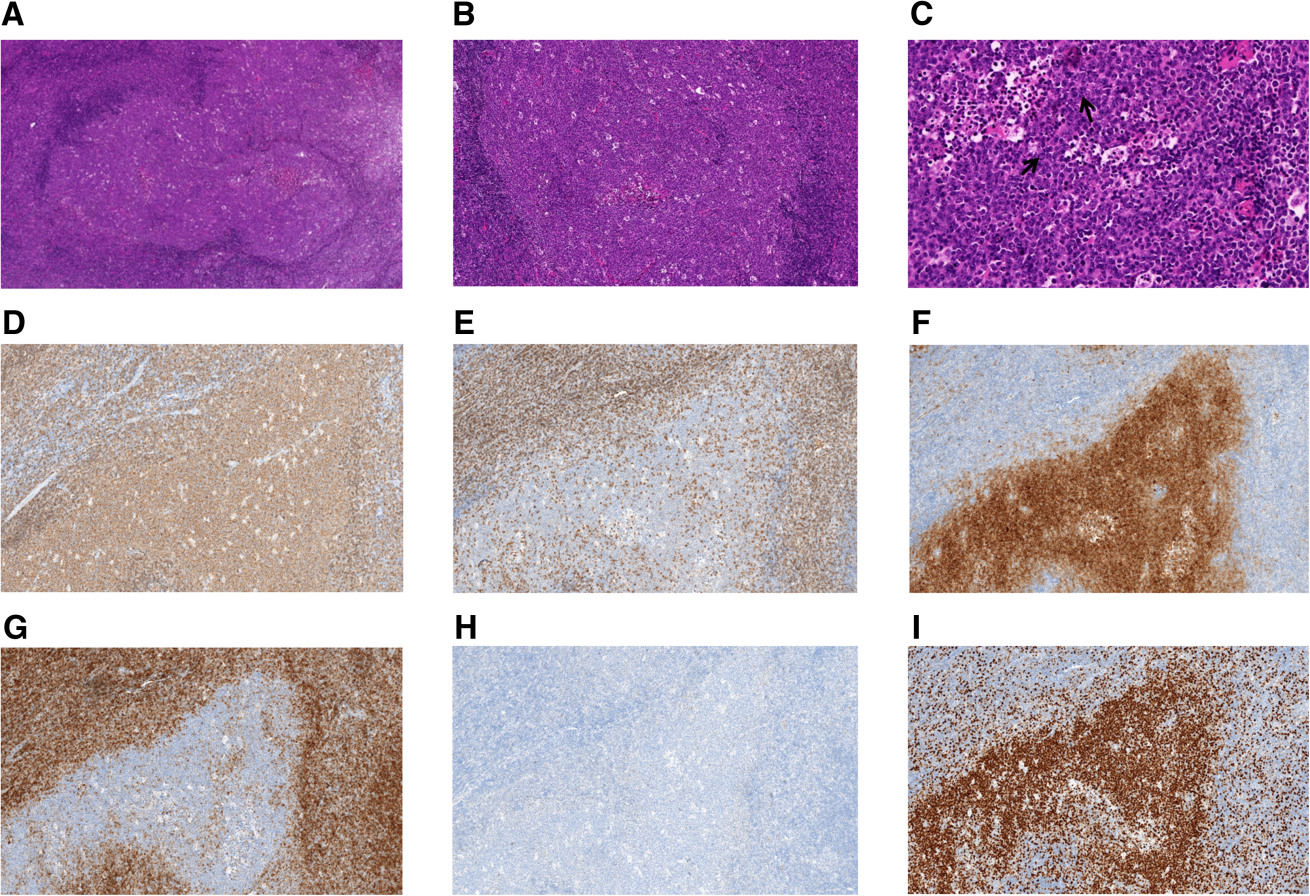
Histologic and immunohistochemical features of pediatric-type follicular lymphoma (Case 2). (**A**) Representative images of hematoxylin and eosin staining. Neoplastic follicles are enlarged, irregular, and creeping, and their mantles are thinned (original magnification, 40×). (**B**) Numerous macrophages with tangible bodies confer a starry sky appearance (original magnification, 100×). (**C**) Lymphoma cells are monotonous, intermediate-sized, and morphologically different from centrocytes or centroblasts in adult FL. The cells are typically blastic in appearance, with dispersed chromatin and inconspicuous nucleoli (original magnification, 400×). ↑: blastoid cells. (**D–I**) Immunohistochemistry of PTFL EnVision (original magnification, 100×). (**D**) CD20, (**E**) CD3, (**F**) CD10, (**G**) BCL2, (**H**) MUM1, and (**I**) Ki-67.

Taking Case 2 as an example, H&E staining showed extensive proliferation of follicular cells. Under a low-power microscope, it was observed that the structure of the lymph nodes was destroyed, and crowded, swollen, and irregular follicle-like structures with different sizes were seen, which were creeping forms (Figure 1A). The “starry sky” pattern was easily observed, normally a feature of reactive follicles caused by tangible body macrophages. The polar distribution of the light and dark areas of the follicles disappeared, and the mantle area became thinner or disappeared in some areas (Figure 1B). At high magnification, neoplastic follicles were found to be composed of uniform, medium-to-large blastoid cells (Figure 1C). In addition, there is no histologic heterogeneity throughout the lymph node. Follicle centers were strongly positive for BCL6 and CD20 (Figure 1D) and negative for BCL2 (Figure 1G), CD3 (Figure 1E), CD5, and CyclinD1. Neoplastic follicles of two cases were positive for CD10 (Figure 1F), and one case was positive for MUM1 (Figure 1H). CD21 showed dilated growth of a follicular dendritic cell network in the neoplastic follicles. In addition, Ki-67 was highly expressed in all four cases (Figure 1I).

### 3.3. FISH and B-cell clonality test

**Figure 2:**
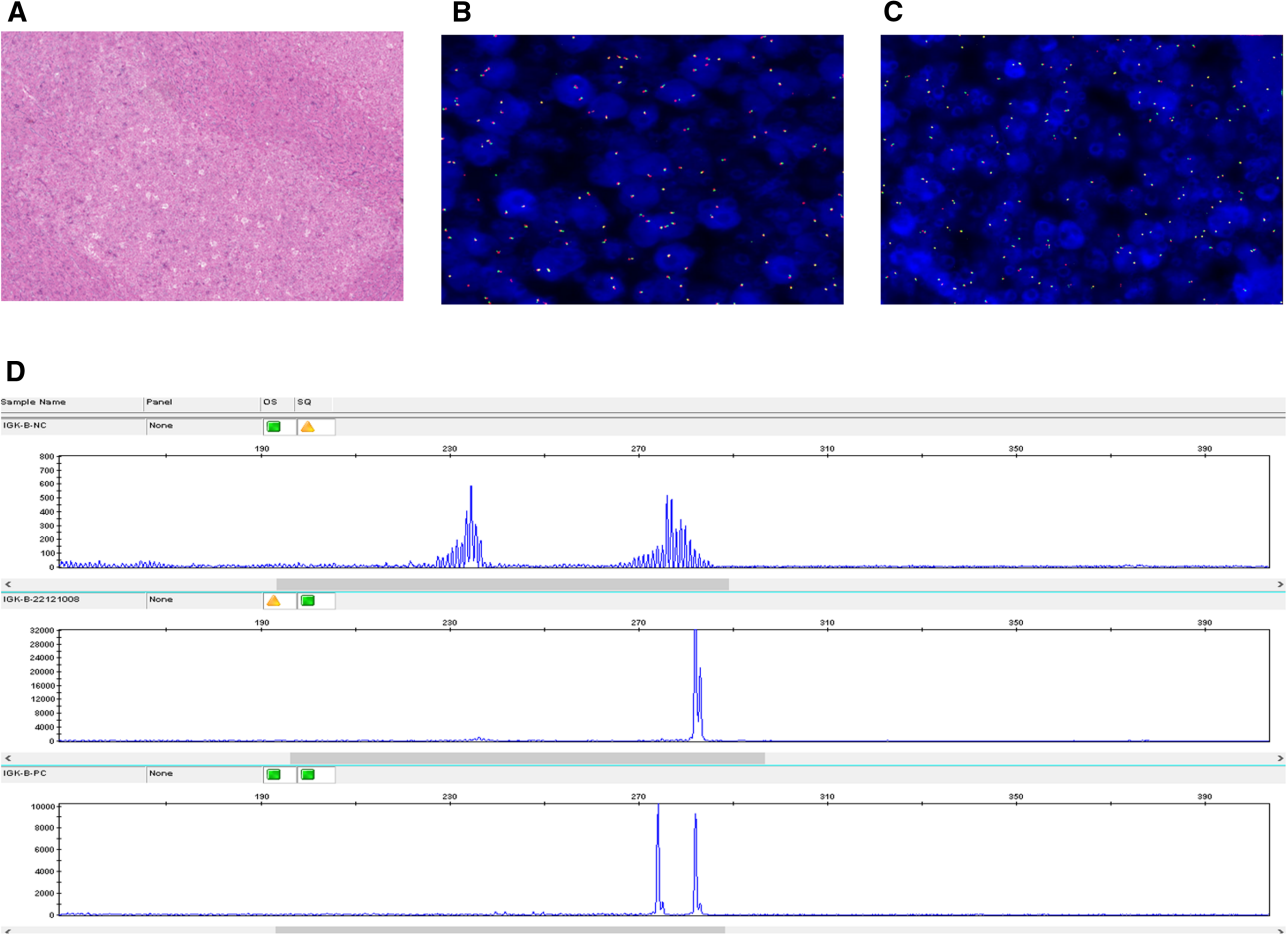
EBER, BCL-2, and B-cell monoclonal rearrangement analysis (Case 2). (**A**) EBER hybridization was negative. (**B**) FISH was negative for *BCL2* rearrangements. (**C**) FISH was negative for *IRF4* rearrangements. (**D**) *IGK* gene rearrangement was positive.

EBER hybridization, *BCL2* gene breaks, and *IRF4* gene breaks were all negative in the four cases (Figures 2A–C), while the *IG* gene rearrangements were all positive (Figure 2D). There were polyclonal backgrounds, but no oligoclonal backgrounds, in the four cases. Products generated from the four diagnostic samples that fall within the valid size range and are at least three times the amplitude of the third largest peak in the polyclonal background are consistent with a positive peak; therefore, the detection of clonal immunoglobulin gene rearrangements was positive.

### 3.4. Mutational landscape

Targeted NGS was performed on all four cases. A total of 15 SNVs or indels were detected in 12 genes, including *PCLO*, *CD79B*, *MYD88*, *GNA13*, *KMT2D*, *TCF3*, *MAP2K1*, *IRF8*, *FOXO1*, *POLE*, *INPP5D*, and *FAT4*. Two of the four cases had *IRF8* gene mutation (Table 2), and one case had a dual mutation of the *MAP2K1* gene.

**Table 2:** Genetic alterations of the four PTFL patients.

Case	Mutated genes	Exons	Nucleobase changes	Amino acid changes	Mutation frequency (%)	Genomic variant categories^a^
1	*PCLO*	exon5	c.8778_8780dupTGA	p. D2926_E2927insD	89.90	I
	*CD79B*	exon5	c.587A>G	p. Y196C	16.70	I
	*MYD88*	exon4	c.728G>A	p. S243N	13.10	I
	*GNA13*	IVS1	c.283+2T>C	—	3.20	I
	*KMT2D*	exon31	c.7478_7479delinsT	p. G2493Vfs*50	66.04	I
2	*TCF3*	exon15	c.1291_1293delinsAGT	p. G431S	46.30	III
	*MAP2K1*	exon2	c.157T>C	p. F53l	12.90	II
	*MAP2K1*	exon2	c.199G>A	p. D67N	1.80	II
	*IRF8*	exon2	c.67T>C	p. Y23H	2.50	III
3	*FOXO1*	exon1	c.285_293delGGCGGCGGC	p. A100_A102del	39.60	I
	*IRF8*	exon3	c.197A>G	p. K66R	2.50	I
4	*MAP2K1*	exon2	c.171G>T	p. Lys57Asn	10.48	II
	*POLE*	exon43	c.5818T>G	p. Ser1910Ale	48.82	III
	*INPP5D*	exon27	c.3430G>T	p. Gly1144Cys	48.01	III
	*FAT4*	exon1	c.4189A>G	p. Arg1397Gly	9.12	III

^a^ AMP/ASCO/CAP guidelines (4): I, variants with strong clinical significance (level A or B evidence); II, variants with potential clinical significance (level C or D evidence); III, variants with unknown clinical significance; IV, variants that are benign or likely benign.

## 4. Discussion

In 1979, Frizzera et al. (5) proposed for the first time that FL in children had unique clinical and morphological characteristics. Subsequently, several studies found that FL in children differs from typical FL in adults in terms of histomorphology, molecular genetics, immunophenotyping, and prognosis. Thus, PTFL was officially recognized as a definitive entity in the updated 2016 WHO classification. PTFL is a rare indolent B-cell lymphoma (2). In this study, we retrospectively analyzed the clinicopathological and genetic features of four PTFL cases and reviewed related pieces of literature.

Although PTFL was initially identified in children, it was subsequently found in young adults and even older patients (6). PTFL exhibits a predominance in men, with a male-to-female ratio ranging from approximately 4:1 to 10:1 (7). The four patients in our study were 6, 18, 13, and 15 years old, respectively, and all were males. PTFL most commonly occurs in the lymph nodes in the head and neck but is less frequent in the inguinal and axillary nodes. The tonsil often occurs outside the node. PTFL can also arise in the liver, spleen, mediastinum, parotid gland, and other parts (8, 9). Our patients presented with enlargement of the right cervical lymph node, left parotid gland mass, and left ear mass. PTFL in the parotid gland area of children is rare. Jangyoun et al. reported one case of PTFL occurring in the left parotid gland region (9), which had similar clinical characteristics to Case 2 in our study. PTFL is a localized disease, often without systemic symptoms; most cases are diagnosed at stage I or II, which can be treated by simple surgical resection. Occasionally, chemotherapy is a feasible option in patients with slightly advanced PTFL that is difficult to resect surgically, with a good prognosis (6, 10). Case 1 was diagnosed with clinical stage III and underwent follow-up for 28 months. No operation or chemotherapy was performed. Case 2 was diagnosed with clinical stage I and underwent left parotidectomy and follow-up for 16 months. Case 3 was diagnosed with clinical stage I and underwent simple resection and follow-up for 12 months. Case 4 was diagnosed with clinical stage I and underwent simple resection and follow-up for 16 months. No disease progression was observed in all cases.

Histopathologically, the lymph node exhibits an effaced architecture and large expansile follicles with attenuated mantle zones and a serpiginous growth pattern. The follicles are non-polarized, but a partial starry sky pattern with tangible body macrophages may be observed. Lymphoma cells are monotonous, intermediate-sized, and morphologically different from centrocytes or centroblasts in adult FL. Lymphoma cells are typically blastic in appearance, with dispersed chromatin and inconspicuous nucleoli. Immunophenotypically, lymphoma cells express germinal centripetal markers such as CD10, BCL6, and HGAL and mature B-cell markers such as CD20, CD79a, and PAX-5 (3, 11). Most cases exhibit a high Ki-67 proliferation index, but not MUM1, CyclinD1, and C-myc. The BCL2 protein is often negative, but a minority of patients present a weak intensity. Neither *t* (4;18) translocation nor *BCL2, BCL6, MYC*, and *IRF4* rearrangements were observed (12, 13). Nevertheless, all PTFL were positive for the *IG* gene rearrangement. Interestingly, the clinical features, pathological morphology, immunophenotype, and molecular genetics of the four patients in this study were consistent with the above characteristics.

With the development of high-throughput sequencing, studies have disclosed that *BCL2* rearrangement and mutations in histone methyltransferases (*KMT2D* and *EZH2*) and acetyltransferases (*CREBBP* and *EP300*) are the hallmarks of FL (12, 14). However, low genomic complexity and frequent aberrations in *TNFRSF14*, *MAP2K1*, and *IRF8* genes characterize PTFL (12, 15, 16). It lacks the *BCL2* gene rearrangement, and mutations in epigenetic modifier genes, including *KMT2D*, *CREBBP*, and *EP300*, are less common. Our study detected 15 mutations in 12 genes. Two cases exhibited *IRF8* gene mutation, and none of the four cases had *TNFRSF14* mutation. According to the guidelines for somatic cell variation interpretation jointly formulated by the American Society for Clinical Pathology (AMP)/American Society of Clinical Oncology (ASCO)/College of American Pathologists (CAP) in 2017, all gene variations in Cases 1 and 3 were of grade I, which has a strong clinical significance in cancer diagnosis, prognosis, and/or therapeutics. *CD79B*, *MYD88*, and *IRF8* were missense mutations. *PCLO* and *KMT2D* were insertion mutations, of which the mutation frequency of *PCLO* was quite high. *FOXO1* was a deletion mutation, while *GNA13* was a splicing mutation in intron 1, leading to abnormal splicing of messenger RNA, thus affecting protein function. The genetic variations in Cases 2 and 4 were of grade III, except for *MAP2K1*, which was classified as grade II. The clinical significance of grade III variants was unclear, while grade II variants (with evidence of Class C or D) had potential clinical significance. In Cases 2 and 4, the *MAP2K1* mutation was repeated within exon 2, which encodes the negative regulatory region domain of the MEK1 protein. *TCF3* were insertion mutations. *POLE*, *INPP5D*, and *FAT4* were missense mutations. At present, there is scarcely any definite conclusion about the mechanism of these rare gene aberrations involved in PTFL, and thus, more prospective studies are needed.

The diagnosis of PTFL is more difficult in clinical practice; therefore, it can easily result in leak diagnosis or misdiagnosis. In addition, the treatment and prognosis of PTFL are quite different from other lymphomas; hence, it should be distinguished from the following diseases:

• Classical FL: it mainly affects adults but rarely occurs in pediatric and young adult populations. Most of the histological grades (approximately 80%) are low grades. The immunophenotypes of FL often express BCL2, BCL6, and CD10. *BCL2* and *BCL6* gene rearrangements characterize the genetics of FL. In addition, FL in adults with negative BCL2 translocation often expresses CD23 and has a high proliferation rate; however, it lacks CD10 expression, which can be differentiated from PTFL (17).
• *IRF4*-rearranged large B-cell lymphoma: like PTFL, it occurs in children and young adults. The head and neck regions, such as Waldeyer's ring and cervical lymph nodes, are the most involved. However, the morphology is characterized by a follicular proliferation of medium-sized blastoid cells without BCL2 translocations and a lack of starry sky phenomena. Tumor cells consistently express IRF4/MUM1 with *IRF4* gene rearrangements (7).
• Pediatric nodal marginal zone lymphoma (PNMZL): it was included as a provisional entity in the revised 4th WHO classification of lymphoid neoplasms. Morphologically, PNMZL often demonstrates large, expanded follicles disrupted by mantle zone cells, resembling progressive transformation of germinal centers. The atypical B cells in PNMZL often co-express CD43 and may be positive for BCL2, while germinal center B-cell markers (CD10 and BCL6) are often negative. Lymphoma cells are negative for Gide, which highlights the expanded mantle zone B cells in PTGC-like follicles. The Ig gene is clonally rearranged. Recent studies have shown that PNMZL and PTFL show overlapping clinicopathological and molecular genetic characteristics. Julia et al. proposed to rename PNMZL as “PTFL with or without marginal zone differentiation” (18).
• Reactive follicular hyperplasia (RFH): the key architectural findings distinguishing RFH from PTFL (lack of nodal effacement, intact polarization of follicles) are often absent based on cytological studies. Lymph nodes with RFH also contain numerous tangible body macrophages in the background, imparting a starry sky appearance, and the immunophenotype is almost consistent with PTFL (19). Recently, Agostinelli et al. (20) showed that the positive rate of FOXP-1 in PTFL was 92%, but it was negative in the reactive germinal center. This discovery led to the emergence of FOXP-1 as a new diagnostic marker for PTFL and helped distinguish RFH.

As with treating PTFL, the United States National Comprehensive Cancer Network (NCCN) guidelines mainly recommend the watch-and-wait strategy for benign tumors. Chemotherapy is no longer the first-line therapy. However, NCCN still recommends radiotherapy or chemotherapy of the affected regions (when the local disease is severe) as an alternative treatment option (21). Overall, PTFL has a good prognosis, with a 5-year survival rate of >95% and a 2-year disease-free survival rate of approximately 94% after chemotherapy for patients with advanced-stage PTFL (22, 23). In conclusion, studying the clinicopathological and genetic mutation landscape of PTFL can expand our understanding of PTFL, thus avoiding leak diagnosis, misdiagnosis, and overtreatment.

## Data Availability

The original contributions presented in the study are included in the article/Supplementary Materials; further inquiries can be directed to the corresponding authors.
